# A Fundamental Study on the Removal of Vascular Pulsation Artifacts Using U-Net-Based Deep Neural Network

**DOI:** 10.7759/cureus.85400

**Published:** 2025-06-05

**Authors:** Tomoko Soma, Norio Hayashi, Yusuke Sato, Toshihiro Ogura, Masumi Uehara, Haruyuki Watanabe, Yoshihiro Kitoh

**Affiliations:** 1 Division of Radiology, Shinshu University Hospital, Matsumoto, JPN; 2 Department of Radiological Technology, Graduate School of Radiological Technology, Gunma Prefectural College of Health Sciences, Maebashi, JPN; 3 Department of Radiological Technology, Gunma Prefectural College of Health Sciences, Maebashi, JPN; 4 Department of Radiology, Gunma University Hospital, Maebashi, JPN

**Keywords:** artifact removal, deep learning, flow artifact, image quality improvement, mri, u-net

## Abstract

Introduction

Artifacts caused by vascular pulsation manifest as periodically high signals in the phase direction, often overlapping the target area and hindering accurate observation. Traditionally, these artifacts have been mitigated using flow compensation and presaturation pulses. However, complete removal remains challenging owing to extended imaging times and the need to consider the specific absorption rate. Therefore, we aimed to propose a deep learning network for postprocessing to reduce these artifacts.

Materials and methods

Following approval from the institutional ethics committee, magnetic resonance imaging scans were conducted on 15 adult volunteers to create an image dataset. Short tau inversion recovery (STIR) images of the lower leg, where artifacts were prevalent, were acquired. The same cross-section was imaged under conditions likely to produce artifacts and conditions designed to minimize artifacts. We propose an artifact reduction network that combines a batch normalization layer and a dropout layer based on the U-Net architecture. The network performance was evaluated using the peak signal-to-noise ratio (PSNR) and structural similarity index measure (SSIM) metrics on the test images. Visual evaluations were conducted using a five-point scale to assess artifact reduction and image resolution. Statistical analyses were performed for each evaluation metric. Profiles of the artifact-prone areas were obtained and assessed before and after artifact reduction.

Results

The average PSNR was 27.83 and 28.57 for the artifact-laden and corrected image groups, respectively. The average SSIM values were 0.869 and 0.882 for the artifact-laden and corrected image groups, respectively. No significant differences were observed between the artifact-laden and corrected image groups for either PSNR (p = 0.315) or SSIM (p = 0.436). The average visual assessment scores for artifact presence were 4.68, 3.52, and 4.34 for the reference, artifact-laden, and corrected image groups, respectively. The average visual assessment scores for image resolution were 4.34, 4.30, and 3.86 for the reference, artifact-laden, and corrected image groups, respectively. No significant differences were observed between the reference and corrected image groups in the presence of artifacts (p = 0.456), although significant differences were noted between these groups and the artifact-laden image group. Furthermore, no significant differences were observed among the three groups regarding resolution evaluation.

Conclusion

To our knowledge, this is the first study to apply deep learning to reduce flow artifacts caused by vascular pulsation using STIR images. We proposed a U-Net-based pulsation artifact reduction network and demonstrated its potential utility. Further detailed evaluation is required to develop an approach suitable for clinical application.

## Introduction

Magnetic resonance imaging (MRI) offers high tissue contrast and is an excellent modality for diagnosing lesions [1, 2]. However, it has certain limitations, such as image quality degradation owing to frequent artifacts and prolonged imaging times [3]. Appropriate parameter settings are crucial, considering the patient's condition and trade-offs between imaging time, signal-to-noise ratio (SNR), and resolution. Consequently, obtaining high-quality artifact-free images within the constraints of limited imaging time is often challenging. "Re-imaging" is one of the most critical issues to avoid to ensure the smooth progression of examinations. To expedite the acquisition of diagnostic-quality images, it is desirable to develop advanced image-processing methods to address image quality degradation.

Recently, numerous studies have demonstrated the effectiveness of deep learning in image processing. Various types of networks, including convolutional neural networks, have been developed and are increasingly being applied in the field of medical imaging. Additionally, there have been reports on networks designed to address MRI artifacts [4-8].

For instance, dynamic liver examinations typically require imaging while the patient holds their breath. However, some patients may be unable to adequately hold their breath, leading to respiratory motion artifacts. Tamada et al [4]. and Zhang et al [5]. developed neural networks for removing respiratory motion artifacts using deep learning techniques. The network proposed by Zhang et al [5] was subsequently tested using clinical data from Kromrey et al [6], who reported a significant improvement in the image quality. Furthermore, there have been studies on networks designed to remove motion artifacts caused by body movements in head images [7] and networks aimed at estimating and correcting distortions owing to metal artifacts [8].

Vascular pulsation is a common artifact observed in magnetic resonance images. This motion artifact is characterized by a high-signal anomaly that consistently repeats at the same distance above or below the pulsating artery and appears as a positional shift in the phase-encoding direction, one of the directions in which signals are filled on MRI. These artifacts are particularly evident in blood vessels on fat-suppressed images of the extremities and in the aorta when using contrast-enhanced or fat-suppressed abdominal images. This prominence is owing to the suppression of fat signals, which enhances the relative visibility of signals from blood vessels and water-containing tissues. These artifacts manifest as false images that overlap with the target or lesion, potentially hindering an accurate diagnosis. Traditionally, this issue has been addressed using flow compensation, a correction method for pulsation and flow artifacts in MRI, along with presaturation pulses, which eliminate the signals flowing into the imaging slice. However, these methods often result in prolonged imaging times and require the consideration of the specific absorption rate, an index used to assess the thermal effects of electromagnetic energy absorption in the human body, thereby making complete artifact removal challenging.

Kim et al [9] reported a successful flow artifact reduction using deep learning. In their study, they employed a Cycle Generative Adversarial Network (CycleGAN) model to investigate the T2 contrast for reducing cerebrospinal fluid (CSF) flow artifacts on cervical spine MRI, achieving notable artifact reduction. No research has been conducted on the removal of flow artifacts caused by vascular pulsation; however, it is believed that a deep-learning approach would be clinically effective, similar to its application in removing CSF-related artifacts. This study aimed to propose a deep learning network for postprocessing to reduce these artifacts.

## Materials and methods

Equipment and dataset

The MRI system used was a 3.0T MAGNETOM Prisma (Siemens Healthineers, Forchheim, Germany), with imaging conducted using a body array coil. MATLAB 2023a (MathWorks, Natick, USA) was used to develop and analyze the deep learning network, and the computations were performed using a GEFORCE GTX 1070 GPU (NVIDIA, Santa Clara, USA). The images were reviewed using a RadiForce RX360 (EIZO Corporation, Ishikawa, Japan). All statistical analyses were performed with EZR (Jichi Medical University, Tochigi, Japan), which is a graphical user interface for R (The R Foundation for Statistical Computing, Vienna, Austria) [10].

To generate the image dataset, MRI scans were performed on 15 adult volunteers, with the approval of the institution’s ethics committee. The ethics application for volunteer imaging was approved in November 2020 and remains valid until November 2025 (Institutional Review Board of the Shinshu University Hospital issued approval no. 4960). All volunteers provided informed consent after being fully briefed on the purpose of the study and the nature of the scans, after which consent forms were obtained.

The imaging parameters were as follows: repetition time (TR), 7000 ms; echo time (TE), 75 ms; inversion time (TI), 220 ms; slice thickness, 3 mm; and field of view (FOV), 220 mm. Short tau inversion recovery (STIR) axial images of the lower leg, which is prone to artifacts, were acquired. Pulsatile blood flow artifacts, which are periodic physiological phenomena, were expected to be reduced through pulse-synchronized imaging. Consequently, STIR images of the same slice were captured under conditions designed to minimize artifacts (flow compensation (+), pre-saturation pulse (+), and pulse synchronization (+)) and conditions likely to induce artifacts (flow compensation (-), pre-saturation pulse (-), and pulse synchronization (-)). Henceforth, the images acquired under artifact-minimizing conditions are referred to as reference images, whereas those captured under artifact-inducing conditions are referred to as artifact images.

The dataset was curated by excluding images deemed inappropriate, such as those with insufficient artifacts. The artifact components were isolated by subtracting the reference image from the corresponding artifact image of the same slice, resulting in a different image. The training dataset comprised 173 pairs of artifact images and their corresponding difference images. Ten pairs of artifact images were used for testing. The trained network estimates the artifact components and generates the estimated images. The corrected images were obtained by subtracting the estimated images from the artifact images.

Network

We developed a variant of the U-Net architecture to remove artifacts, as previously reported [11]. This network integrates three key components: the U-Net [12], batch normalization (BN) layer [13], and dropout layer [14]. The modified network effectively reduced motion artifacts in magnetic resonance images. The network is designed as a residual network that estimates only the artifact components from the artifact images. Given that vascular pulsatile flow artifacts, which are similar to motion artifacts, appear periodically in the phase-encoding direction, we hypothesized that this network would be similarly effective. Among the three network configurations evaluated in the previous study, the most effective configuration was selected for this study.

**Figure 1:**
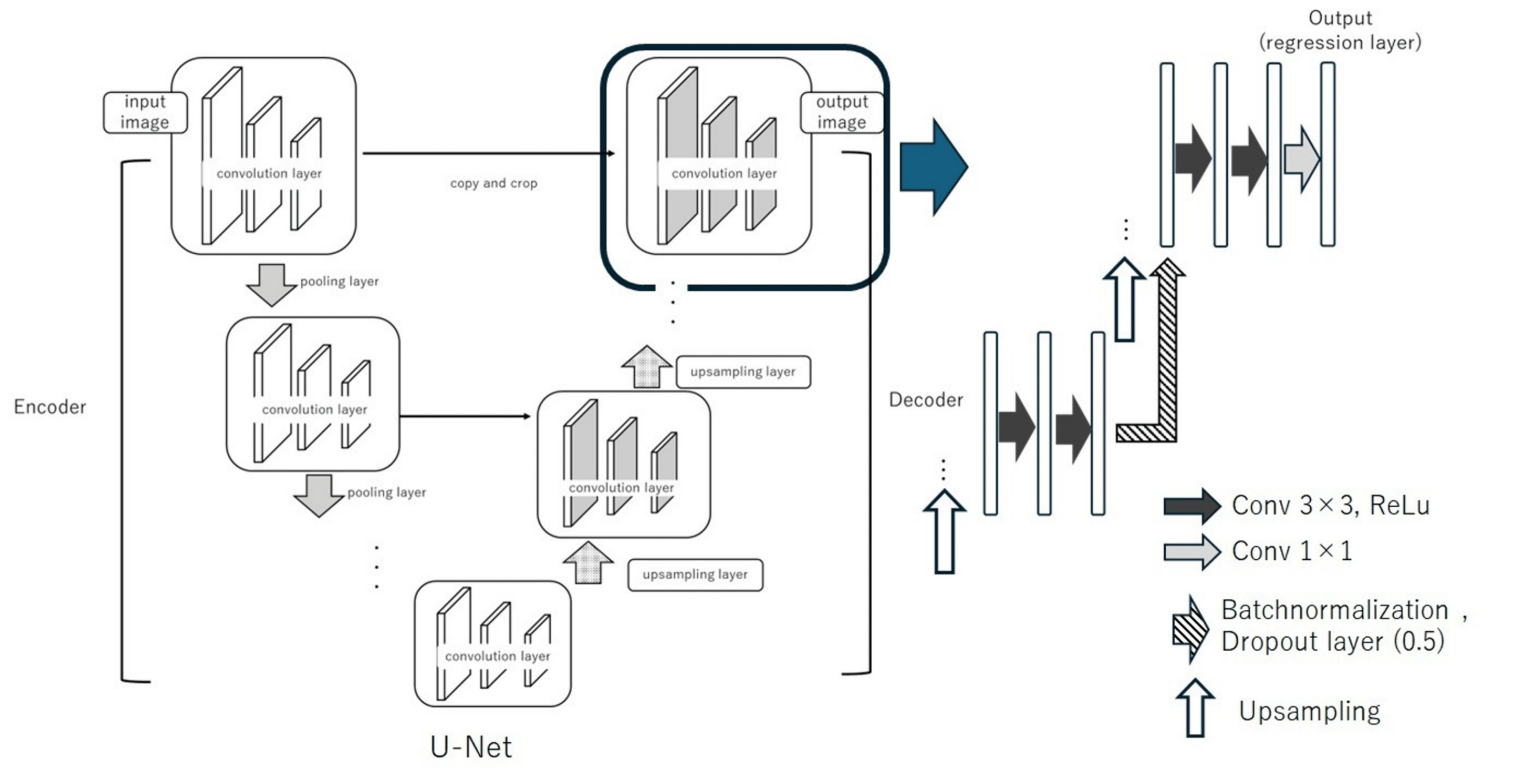
Architecture of the U-Net variant used for artifact removal, incorporating dropout and batch normalization layers We developed a variant of the U-Net architecture to remove artifacts, as previously reported. Figure 1 shows the architecture that proved most effective in our previous study, which effectively incorporates dropout and BN layers into the U-Net. Source: Maruyama et al [11]. Available under a Creative Commons Attribution-NonCommercial-NoDerivatives 4.0 International License (https://creativecommons.org/licenses/by-nc-nd/4.0/). The figure has not been modified.

An overview of the network architecture is as follows: the base structure is U-Net, which was originally proposed in 2015 for medical image segmentation. U-Net features an encoder-decoder architecture and has been increasingly used for segmentation and image generation tasks [4, 15]. To enhance convergence, BN and dropout layers were added after the final rectified linear unit (ReLU) function in the U-Net decoder. BN layers are typically applied before and after the activation functions within the model. When used with the dropout layers, the dropout layer must follow the BN layer to prevent accuracy degradation [16]. The BN layer mitigates the gradient vanishing problem caused by bias, thereby preventing learning stagnation and the loss of expressive power. This was achieved by adjusting the initial weight values and normalizing the activations to fit a Gaussian distribution with a mean of 0 and a variance of 1. The dropout layer, which is a regularization technique, prevents overfitting by randomly deactivating nodes during training. This approach significantly accelerates the training of deep-learning models and enhances the efficiency of processing large datasets. A schematic of the network architecture is shown in Figure 1 [11].

Learning parameters

The training conditions were as follows: Adam optimizer with a loss function, initial learning rate of 0.001, mini-batch size of 12, and a maximum number of 200 epochs. Additionally, early stopping was implemented, whereby the training was automatically terminated if the validation root-mean-square error (RMSE), evaluated every five epochs, failed to improve in five consecutive evaluations. The training process was repeated five times, and the performance was assessed accordingly.

Statistics and evaluation

Ten images were used for testing. Before and after the artifact correction, we measured the structural similarity index measure (SSIM) and peak signal-to-noise ratio (PSNR) with respect to the reference images. The PSNR and SSIM were calculated using the central 120 pixels, where flow artifacts were expected to be present, while excluding the 100 pixels at each end. The SSIM and PSNR are expressed in Equations 1 and 2.

By representing the pixel values of image X by *x*, the pixel values of image Y by *y*, the mean pixel value of image X by *μ_x_*, the mean pixel value of image Y by *μ_y_*, the standard deviation of the pixel values of image X by *σ_x_*, the standard deviation of the pixel values of image Y by *σ_y_*, the covariance of pixel values between images X and Y by *σ_xy_*, and employing *C_1_* and *C_2_* as constants to stabilize the output values, we get 

\begin{array}{rcl} SSIM(x,y) & = & \frac{(2\mu_{x}\mu_{y}+C_{1})(2\sigma_{xy}+C_{2})}{(\mu_{x}^2+\mu_{y}^2+C_{1})(\sigma_{x}^2+\sigma_{y}^2+C_{2})} \cdots (1)\\ \end{array}

By representing the maximum pixel value by *R* and the mean squared error by *MSE*, we obtain 

\begin{array}{rcl} PSNR & = & 10\log_{10}\frac{R^{2}}{MSE}\cdots (2)\\ \end{array}

The average SSIM and PSNR values across the five training runs were calculated. These values were compared between the artifact and estimated images using the Mann-Whitney U test, with the significance level set at p < 0.05. Additionally, the artifact region profiles were plotted to evaluate the effectiveness of the artifact removal.

Five radiologists with two to 16 years of experience conducted the visual evaluation. Ten references, artifacts, and corrected images were randomly displayed on an image-reading monitor. Two criteria were assessed: the presence of flow artifacts and image resolution, both rated on a five-point scale [4, 17]. The scoring criteria for the presence of flow artifacts were as follows: 1 = Significant artifacts were observed, rendering the image unsuitable for diagnosis; 2 = Artifacts were present and potentially impacted the diagnosis; 3 = Artifacts were observed, but the image remained diagnosable; 4 = Minor artifacts were present but not affecting the diagnosis; 5 = No artifacts were observed.

For image resolution, the following criteria were considered: 1 = The image was generally unclear and inappropriate for diagnosis; 2 = The image clarity was insufficient and potentially affected the diagnosis; 3 = The image was diagnosable despite some clarity issues; 4 = Some areas were unclear, but the overall image did not affect the diagnosis; 5 = Overall, the image is clear.

The scores from the five radiologists were averaged and analyzed using the Kruskal-Wallis test (p < 0.05), followed by the Steel-Dwass post hoc test.

The results of the statistical analysis were presented using box plots. In the box plots, the central line represents the median. The box spans from the first quartile (Q1) to the third quartile (Q3), and the whiskers extend to the minimum and maximum values within 1.5 times the interquartile range (IQR).

## Results

**Figure 2:**
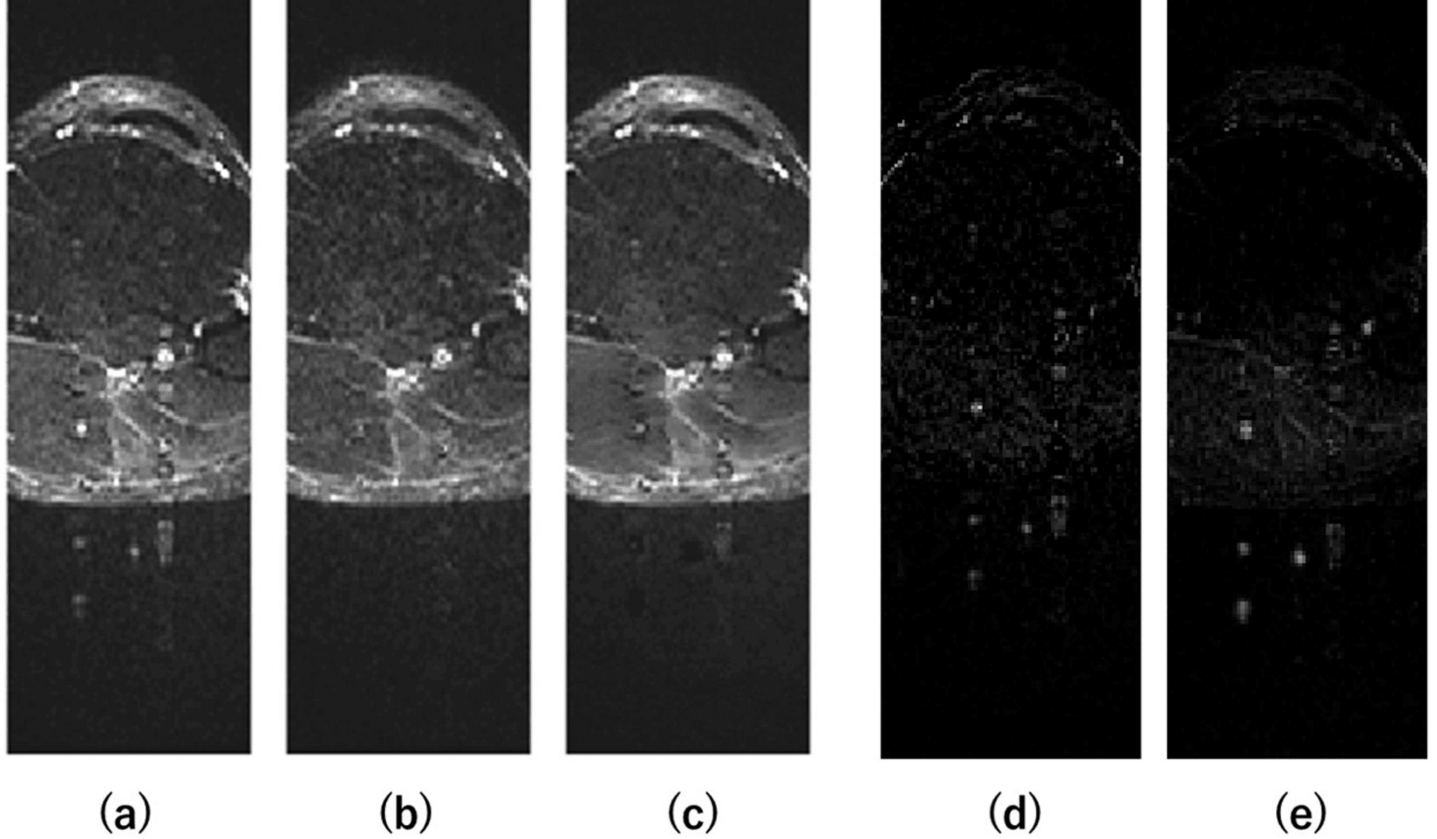
Examples of dataset images and images after flow artifact correction (a) Image with flow artifacts. (b) Reference image with minimal artifacts. (c) Corrected image obtained by subtracting the estimated artifact component (e) from (a). (d) Difference image between (a) and (b). (e) Estimated artifact component generated by the model. Source: Authors.

Figure 2 shows an example of an image following flow artifact correction. Panel (a) shows the artifact image; panel (b) shows the reference image obtained under conditions designed to minimize artifacts; panel (c) depicts the corrected image derived by subtracting the estimated artifact component (e) from panel (a); panel (d) illustrates the difference image created by subtracting panel (b) from panel (a); and panel (e) shows the estimated artifact component. Conversely, (a) and (d) are learned as a pair, the result is estimated as (e), and the corrected image (c) is obtained by subtracting (e) from (a).

**Figure 3:**
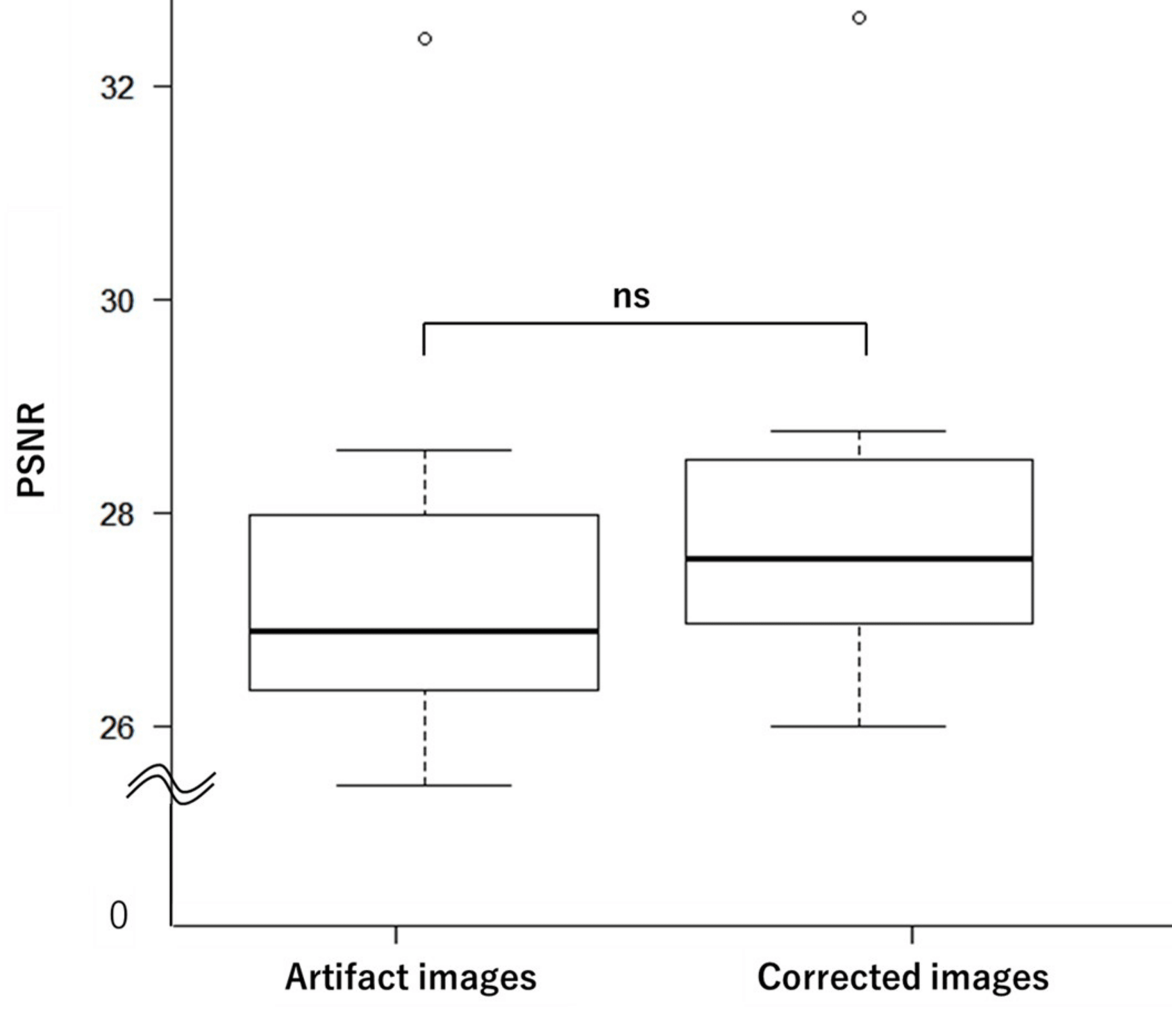
Box plots of PSNR values for artifact and corrected images Statistical analysis revealed no significant difference between the artifact and corrected image groups (p = 0.315). PSNR: peak signal-to-noise ratio; ns: not significant

**Figure 4:**
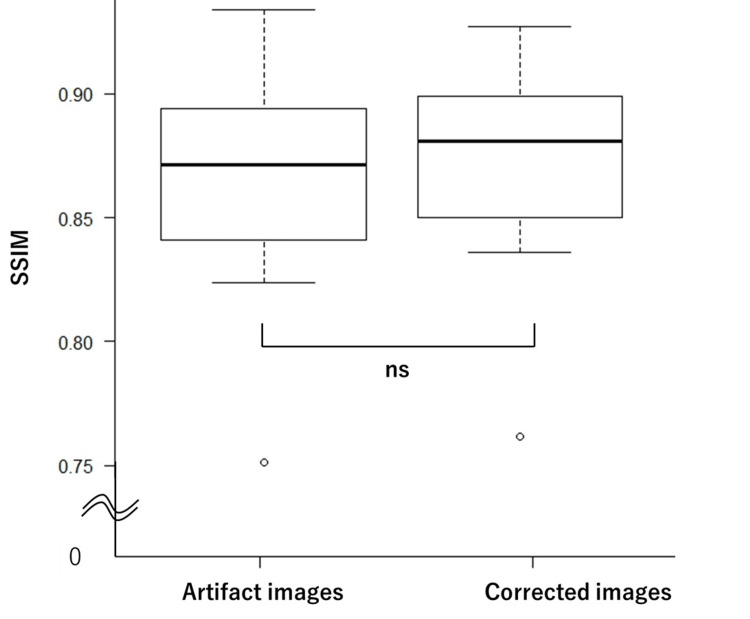
Box plots of SSIM values for artifact and corrected images No significant difference was observed between the two groups in terms of SSIM (p = 0.436). SSIM:  structural similarity index measure; ns: not significant

The average PSNR values were 27.83 and 28.57 for the artifact and corrected image groups, respectively. The average SSIM values were 0.869 and 0.882 for the artifact and corrected image groups, respectively. Box plots illustrating the test results for the PSNR and SSIM are shown in Figure 3 and Figure 4, respectively. The statistical analysis revealed no significant differences between the artifact and the corrected image groups for either PSNR (p = 0.315) or SSIM (p = 0.436).

**Figure 5:**
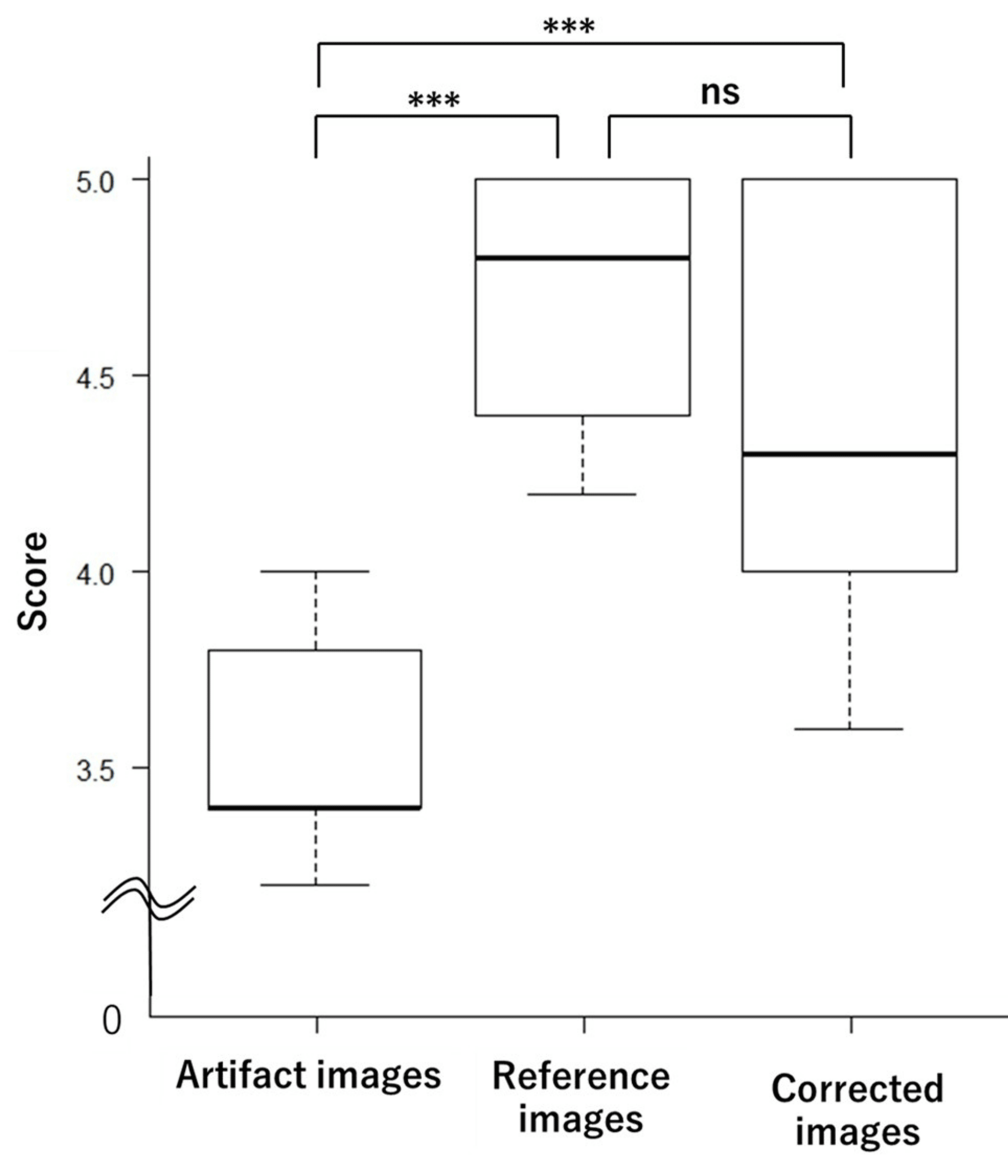
Box plots of visual assessment scores for artifact presence The corrected and reference image groups showed no significant difference (p = 0.456), whereas both differed significantly from the artifact image group. ***p-value < 0.01; ns: not significant

**Figure 6:**
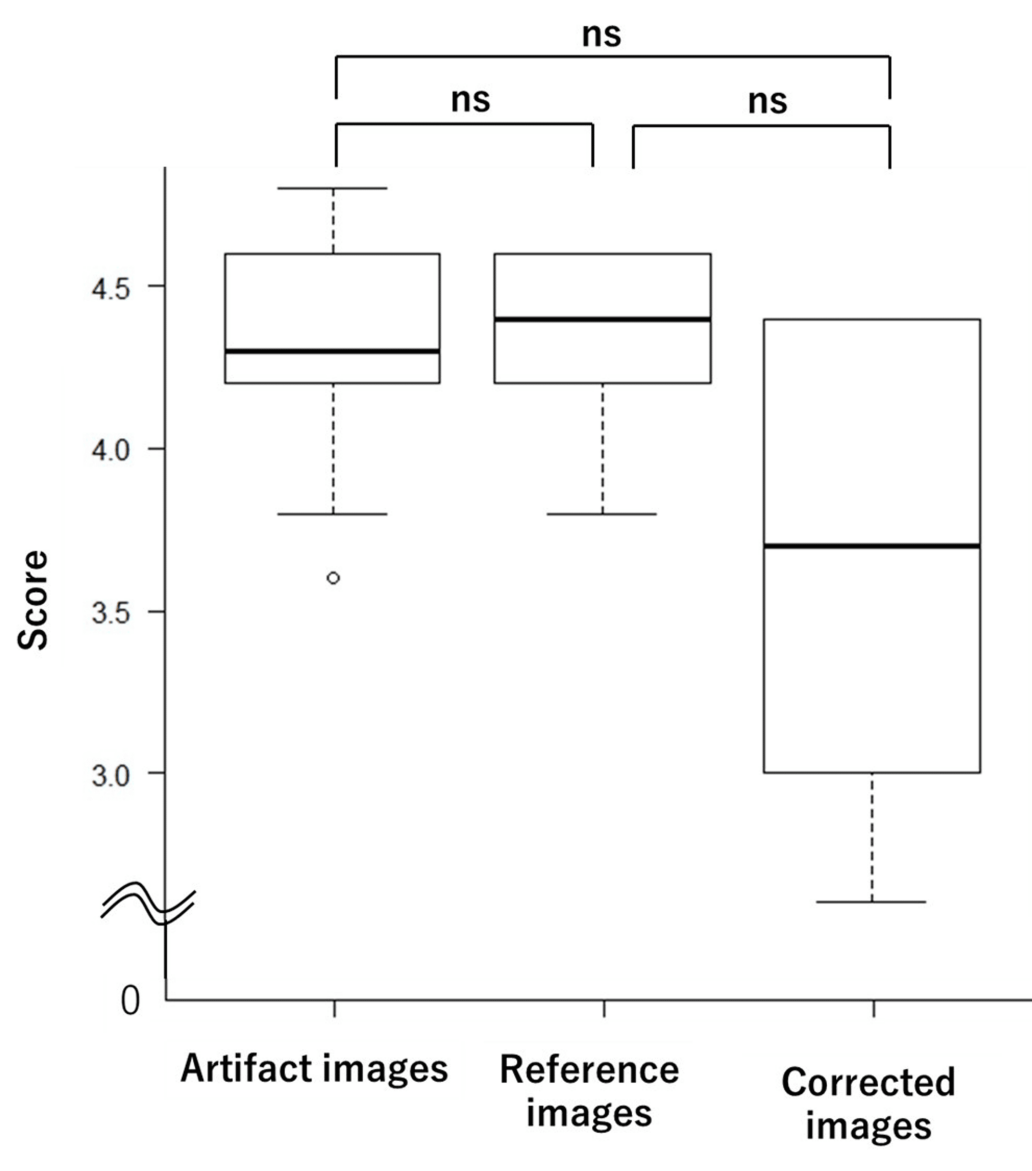
Box plots of visual assessment scores for image resolution No significant differences were observed among the three groups in terms of resolution. ns: not significant

The average scores for the visual assessment of artifact presence were 4.68, 3.52, and 4.34 for the reference, artifact, and corrected image groups, respectively. For image resolution, the average scores were 4.34, 4.30, and 3.86 for the reference, artifact, and corrected image groups, respectively. The scores for each of the 10 test images are shown in Table 1 for artifacts and Table 2 for image resolution. Box plots for these assessments are shown in Figure 5 (artifact presence) and Figure 6 (image resolution). The results indicated no significant difference in artifact presence between the reference and corrected image groups (p = 0.456), although both groups showed significant differences compared to the artifact image group. Additionally, no significant differences were observed in resolution assessments across the three groups.

**Table 1:** The mean scores of the visual assessment for the artifacts The artifact scores (averaged across five raters) for the 10 test images in each image group are shown.

Test image number	Artifact images	Reference images	Corrected images
1	3.8	4.6	4.6
2	3.6	4.8	4
3	3.4	4.4	4
4	3.8	4.2	3.8
5	4	4.8	4.6
6	3.4	5	4
7	3.2	4.2	4.8
8	3.2	4.8	5
9	3.4	5	4.8
10	3.4	5	3.8

**Table 2:** The mean scores of the visual assessment for the image resolution The image resolution scores (averaged across five raters) for the 10 test images in each image group are shown.

Test image number	Artifact images	Reference images	Corrected images
1	3.6	4.4	3.4
2	4.2	3.8	3
3	3.8	4.4	4.4
4	4.2	4.6	3.2
5	4.2	4.4	3
6	4.8	4.2	4.4
7	4.6	4.2	4.4
8	4.6	4.2	4
9	4.6	4.6	4.4
10	4.8	4.2	4.4

**Figure 7:**
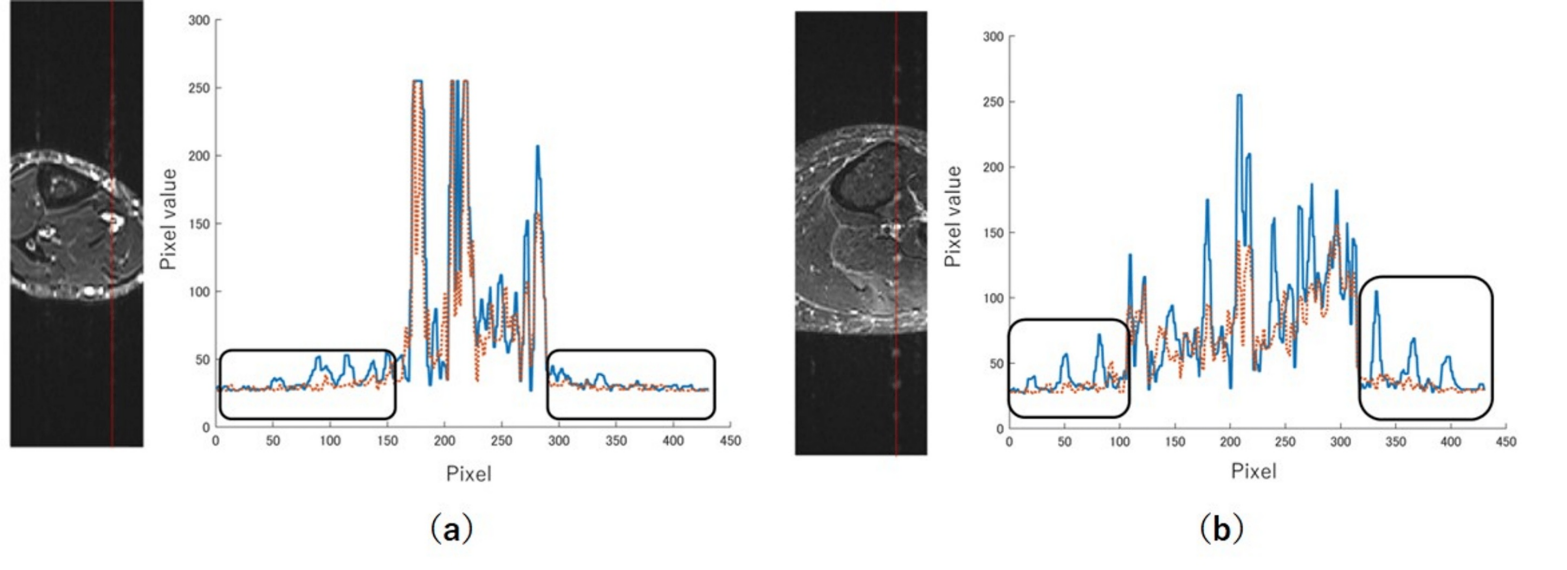
Profiles of flow artifacts in selected regions of interest Red lines on the images indicate the analyzed regions. Solid lines represent the intensity profiles from artifact images, and dotted lines represent those from corrected images. The regions enclosed by the black squares represent the signals from the flow artifacts. The corrected image in panel (a) shows only the removal of the artifact component, whereas in panel (b), the overall signal of the image is reduced.

Figure 7 shows examples of profiles from regions of the artifact and corrected images where the flow artifacts were prominent. The artifact-related signal waves were eliminated from the corrected image curves. However, Figure 7b shows an overall reduction in the signals outside the black square region.

## Discussion

Several networks for motion artifact reduction have been previously compared. In this study, we employed a U-Net-based architecture, which has demonstrated strong performance in prior research [11]. By incorporating BN and dropout layers at the final stage of each decoder, we significantly improved artifact removal performance with only minimal architectural modifications. Since flow artifacts appear periodically along the phase-encoding direction, they can be regarded as a type of motion artifact. Therefore, we applied the network that had shown the best performance in previous studies to the current task.

The trend of artifact reduction shown in Figure 2 was consistent across all 10 test images, with effective suppression and minimal estimation error. Despite these visual improvements, no significant differences were observed in PSNR and SSIM values. This lack of difference is likely because the primary distinction between the artifact and corrected images lies in the presence or absence of flow artifacts, while most of the image structure remains unchanged. This limited the impact on the overall PSNR and SSIM metrics. From a different perspective, this result suggests that the corrected image does not significantly change the overall structure of the image except for artifact areas.

In the visual evaluation of artifact presence, significant differences were observed between the artifact image group and both the reference and corrected image groups, confirming the effectiveness of the network in artifact reduction. In this study, the physical image quality metrics such as PSNR and SSIM may not always correlate with the degree of improvement perceived through visual assessment. Visual evaluation often holds the greatest significance in medical imaging research, as the final interpretation and diagnosis of the images are performed by physicians. This subjective assessment directly reflects the clinical utility and practical applicability. Visual evaluation of the resolution revealed no significant differences among the three groups. This finding suggests that the image resolution was preserved following the correction. However, the corrected image group scored lower in resolution than the other two groups, with greater variance. This variance suggests that the perception of resolution in corrected images may differ among evaluators and may be influenced by their level of experience.

The creation of the corrected images involved subtracting the estimated artifact components from the artifact images. If the estimated component includes subtle anatomical edges or signal variations unrelated to artifacts, the perceived resolution can be reduced. In Figure 7a, only the artifact component appears to have been subtracted, with the signal from normal tissue well preserved. In contrast, Figure 7b suggests that signal reduction may have occurred in tissues beyond the artifact itself, indicating the need for careful interpretation.

We compared our network with those reported in other previous studies. U-Net-based networks continue to demonstrate high performance. For instance, Safari et al [18] developed a custom network for motion artifact reduction and compared its performance with supervised U-Net, CycleGAN, and Pix2Pix models. Although their proposed network achieved excellent accuracy, the supervised U-Net model maintained comparable performance, particularly for minor motion artifacts, where it demonstrated robust results.

Velayudham et al [19] proposed the Transformer-based U-Net Image Prior Generator for denoising brain MRI images. This network delivered high accuracy and offered fast execution time, which was highlighted as an advantage. Our proposed network exhibited similar benefits, offering effective artifact reduction and fast processing. Furthermore, the simpler architecture of this network compared to that of CycleGAN may facilitate artifact estimation, even when trained with smaller datasets [9]. The most significant advantage of the proposed approach is its ability to reduce artifacts through post-acquisition network processing, thereby eliminating the need for additional image sequences that could prolong examination time. This benefit aligns with findings by Kim et al. [9], who used CycleGAN to reduce CSF-related artifacts.

This study has some limitations. The number of training images was small; it focused only on STIR lower-leg images and did not consider using contrast agents, which are known contributors to flow artifacts. Additionally, since only volunteer images were used, the dataset lacked pathological diversity, preventing assessment in a clinical setting. In addition, the limited size of the dataset may have hindered improvements in PSNR and SSIM. Expanding the dataset could potentially enhance the performance of these quantitative metrics.

Another limitation is the inter-observer variability in resolution assessments among radiologists. Many previous studies using deep learning for noise or artifact removal have noted the difficulty in preserving edge information and fine structural details [20-22]. The observed decrease in resolution and signal intensity may affect lesion detectability. Further studies are needed to determine whether these limitations negatively impact diagnostic reliability.

The findings of this study highlight the need for improved methods, such as incorporating a resolution enhancement network after artifact reduction, to achieve consistently high-resolution evaluations. Although the target images in this study were limited to the lower leg, the proposed method may be applicable to other anatomical regions in the future. For instance, the abdominal aorta is a common source of prominent flow artifacts, and the approach presented in this study may prove effective in such cases. Further studies are required to demonstrate the broader applicability and clinical utility of this method.

## Conclusions

This study represents the first attempt to use deep learning for the reduction of flow artifacts induced by vascular pulsation. The proposed U-Net-based network showed promising performance in suppressing artifacts while preserving image quality in visual assessment. Although this study focused on STIR images, further research involving larger datasets, such as images with varying contrasts and those obtained from different anatomical regions, is necessary to evaluate the potential for clinical application.
